# OPTICAL IMAGING OF LIPOPOLYSACCHARIDE-INDUCED OXIDATIVE STRESS IN ACUTE LUNG INJURY FROM HYPEROXIA AND SEPSIS

**DOI:** 10.1142/S179354581350017X

**Published:** 2013-06-18

**Authors:** REYHANEH SEPEHR, SAID H. AUDI, SEPIDEH MALEKI, KEVIN STANISZEWSKI, ANNIE L. EIS, GIRIJA G. KONDURI, MAHSA RANJI

**Affiliations:** Biophotonics Laboratory, Department of Electrical Engineering and Computer Science, University of Wisconsin Milwaukee 3200 N Cramer St., Milwaukee, WI 53211, USA; Department of Biomedical Engineering, Marquette University, 1515 W Wisconsin Avenue Milwaukee, WI 53233, USA; Biophotonics Laboratory, Department of Electrical Engineering and Computer Science, University of Wisconsin Milwaukee 3200 N Cramer St., Milwaukee, WI 53211, USA; Biophotonics Laboratory, Department of Electrical Engineering and Computer Science, University of Wisconsin Milwaukee 3200 N Cramer St., Milwaukee, WI 53211, USA; Department of Pediatrics, Cardiovascular Research Center Medical College of Wisconsin, 8701 Watertown Plank Rd Milwaukee, WI 53226, USA; Department of Pediatrics, Cardiovascular Center and Children’s Research Institute, Medical College of Wisconsin CCC, Ste C410, 999 N92 St, Milwaukee, WI 53226, USA; Biophotonics Laboratory, Department of Electrical Engineering and Computer Science, University of Wisconsin Milwaukee 3200 N Cramer St., Milwaukee, WI 53211, USA

**Keywords:** Fluorescence imaging, NADH, FAD, LPS, Hyperoxia

## Abstract

Reactive oxygen species (ROS) have been implicated in the pathogenesis of many acute and chronic pulmonary disorders such as acute lung injury (ALI) in adults and bronchopulmonary dysplasia (BPD) in premature infants. Bacterial infection and oxygen toxicity, which result in pulmonary vascular endothelial injury, contribute to impaired vascular growth and alveolar simplification seen in the lungs of premature infants with BPD. Hyperoxia induces ALI, reduces cell proliferation, causes DNA damage and promotes cell death by causing mitochondrial dysfunction. The objective of this study was to use an optical imaging technique to evaluate the variations in fluorescence intensities of the auto-fluorescent mitochondrial metabolic coenzymes, NADH and FAD in four different groups of rats. The ratio of these fluorescence signals (NADH/FAD), referred to as NADH redox ratio (NADH RR) has been used as an indicator of tissue metabolism in injuries. Here, we investigated whether the changes in metabolic state can be used as a marker of oxidative stress caused by hyperoxia and bacterial lipopolysaccharide (LPS) exposure in neonatal rat lungs. We examined the tissue redox states of lungs from four groups of rat pups: normoxic (21% O_2_) pups, hyperoxic (90% O_2_) pups, pups treated with LPS (normoxic + LPS), and pups treated with LPS and hyperoxia (hyperoxic + LPS). Our results show that hyperoxia oxidized the respiratory chain as reflected by a ~31% decrease in lung tissue NADH RR as compared to that for normoxic lungs. LPS treatment alone or with hyperoxia had no significant effect on lung tissue NADH RR as compared to that for normoxic or hyperoxic lungs, respectively. Thus, NADH RR serves as a quantitative marker of oxidative stress level in lung injury caused by two clinically important conditions: hyperoxia and LPS exposure.

## 1. Introduction

Bronchopulmonary dysplasia (BPD) is a chronic lung condition that affects premature infants who receive supplemental oxygen (hyperoxia) or ventilator support for long periods of time. Studies have shown that the premature lung can be acutely injured by either oxygen or mechanical ventilation, resulting in interference with or inhibition of lung alveolar and vascular development.^1–3^ Premature infants are also more likely to be exposed to infection *in utero* or during postnatal life, which accelerates the subsequent development of BPD. Lipopolysaccharide (LPS) is an endotoxin, which is derived from the cell wall of gram-negative bacteria and induces the release of cytokines and reactive oxygen species (ROS) such as superoxide, hydroxyl radicals and peroxynitrite. The endothelial injury caused by LPS stimulation contributes to vascular remodeling by inhibiting endothelial cell proliferation, migration and angiogenesis, which together contribute to impaired lung growth in BPD. We investigated the hypothesis that the effects of LPS on the immature lung are amplified when exposed to hyperoxic conditions during postnatal life compared to the hypoxic *in utero* environment of the fetus.^4–7^

Acute respiratory distress syndrome (ARDS), a manifestation of acute lung injury (ALI), is a serious illness associated with severe and diffuse injury to the alveolar-capillary membrane of adult lungs. In ARDS, organs are deprived of the required amount of O_2_, which impairs their proper function. Despite the high morbidity and mortality rates of this illness, mechanisms underlying the development of ARDS/ALI remain incompletely understood.^8–11^ Respiratory distress syndrome (RDS) in neonates occurs due to surfactant deficiency and immaturity of the lung parenchyma and vasculature. The course of RDS is characterized by parenchymal lung injury, which leads to impaired gas exchange, neutrophil accumulation in the lung, the expression of pro-inflammatory mediators, increased vascular permeability and damage to the lung epithelium and endothelium. One of the contributing factors in the development of ALI in premature babies is exposure to bacterial infection, including endotoxin from gram-negative bacteria.^8–10,12–14^ Exposure to supplemental oxygen, which is often used in the treatment of RDS, can also contribute to lung injury.

Use of supplemental O_2_ in patients who suffer from hypoxemia and RDS is often life saving since it is necessary to restore blood pO_2_ to a level that supports the metabolic requirements of vital organs. However, prolonged exposure to higher concentrations of O_2_ or hyperoxia leads to enhanced production of ROS and lung injury at the cellular level associated with mitochondrial dysfunction, decreased cell proliferation, DNA damage and alveolar epithelial and endothelial cell death.^15–20^

Neonatal rat pups have been extensively used as a model to study the effects of hyperoxia on lung injury and growth. Previous studies have demonstrated an arrest of lung alveolar and vascular growth with the exposure of neonatal rat lungs to hyperoxia for a period of 7–10 days.^21^ Since rat pups at birth have lung development at the saccular stage similar to premature babies,^22^ the disruption of development by hyperoxia parallels the changes seen in premature babies with BPD. We therefore, selected this model to investigate mitochondrial redox state during hyperoxia induced lung injury.

As previously reported, LPS induces elevated ROS levels in endothelial cells. Decreased anti-oxidant capacity of pulmonary vascular tissue along with increased production of ROS contributes to the injury seen in LPS-induced ALI/BPD. ^7,9,23–27^ ROS generated by LPS exposure in endothelial cells is regarded as a key to the modulation of the pulmonary vascular endothelial damage, which leads to higher oxidative stress. Increased ROS may cause cell injury, activate the inflammatory response, promote cytotoxicity and activate signaling pathways that lead to pro-apoptotic signaling. Thus, LPS has been considered as the principal component in the induction of ALI and BPD in adults and premature infants, respectively.^7,12,28,29^

Fluorescence imaging provides specific information on tissue oxidative stress using intrinsic fluorophores or exogenous tagged proteins. Fluorescence-based techniques have the potential to diagnose tissue metabolic states in intact organs. These techniques are widely used in biomedical applications and have been shown to have high sensitivity and specificity for discriminating between diseased and nondiseased tissues.^20,30–33^ In this study, we are focusing on ROS as the final causative molecule in the pathogenesis of lung injury caused by LPS or exposure to higher O_2_ concentrations. Although ROS generation after exposure of cells to LPS is well documented by *in vitro* investigations, *in situ* evidence is, so far, lacking.^7–9,23,34^ Using optical cryoimaging, we investigated *in situ* ROS detection in intact rat lungs treated with higher O_2_ concentrations and LPS.

Mitochondrial metabolic coenzymes nicotinamide adenine dinucleotide (NADH) and flavoprotein adenine dinucleotide (FADH_2_) are two of the primary electron carriers in oxidative phosphorylation. NADH and FAD (the oxidized form of FADH_2_) are autofluorescent and can be monitored without exogenous labels through the use of optical techniques. These coenzymes are significant in that NADH is primarily fluorescent in its reduced biochemical state, whereas FAD is only fluorescent in its oxidized form. Therefore, by imaging these two coenzymes, we can probe the oxidative state of the metabolism in tissue. The fluorescent signals of these intrinsic fluorophores have been used as indicators of tissue metabolism in injury due to hypoxia, ischemia and cell death.^35–41^ In addition, by evaluating the ratio of these two coenzymes, some of the confounding effects from absorbers, including hemoglobin and collagen, as well as scattering effects can be eliminated as factors undermining the measurement of this oxidative state. Our studies have demonstrated that the ratio of these fluorophores, (NADH/FAD), called the NADH redox ratio (NADH RR), acts as a novel marker of the mitochondrial redox and metabolic state of tissue *ex vivo*^42^ and *in vivo*.^46^ Although this ratio is not a direct measure of the concentrations of these fluorophores, the fluorescence intensity measured is a relative measure of their concentrations.^33^

The primary objective of the present study was to investigate the effect of LPS stimulation on the mitochondrial redox state of normoxic and hyperoxic rat lung models using fluorescence imaging. Using NADH RR, we quantified the effect of LPS on normal versus hyperoxic lungs.

## 2. Materials and Methods

### 2.1. Lung preparation

Neonatal rats were randomly assigned at birth to either postnatal normoxia (21%) or hyperoxia exposure (90%) from birth to 10 days of postnatal age. Each group was further subdivided to intra-peritoneal (IP) LPS injection (10 mg) on postnatal day 7, or to IP injection of equal volume of saline. A separate group of normoxic rat pups were used as controls to isolate and perfuse the lungs with potassium cyanide (KCN) to inhibit the mitochondrial respiratory chain. This study design yielded five different groups of rats consisting of normoxia +/− LPS and hyperoxia +/− LPS and KCN perfused lungs to verify the upper limit of the respiratory chain reduction as described below.

To demonstrate the ability of NADH RR to detect a change in redox state of the electron transport chain, lungs of anesthetized normoxic pups were isolated and perfused *ex vivo* with perfusate (Krebs Ringer bicarbonate solution containing 3% bovine serum albumin) containing 2 mM KCN, which is an inhibitor of mitochondrial complex IV. KCN would be expected to reduce the electron transport chain and as a result, increase NADH RR when compared to that for lungs of normoxic pups. The perfusate was pumped into the lungs at a flow rate of 1 mL/min for 10 min. During this period, the lungs were ventilated (15% O_2_, 6% CO_2_, balance N_2_) at 80 breaths/min. At the end of the perfusion period, the lungs were rapidly frozen for cryoimaging as described below.

#### Hyperoxia exposure

Rat pups were reared from birth in a plexiglass chamber with air and oxygen flow at a sufficient rate (3 L/min) to prevent CO_2_ accumulation in the chamber. Openings in the chamber at the top allowed ambient air in the chamber to be replaced with fresh flow of gas. Air and oxygen were mixed to maintain an oxygen (O_2_) concentration of 90% inside the chamber. Ambient temperature was kept at 27 ± 1°C in the chamber. The chamber was large enough to house the mother and pups for 10 days. Since adult rats are more vulnerable to oxygen injury, mothers were taken out of the chamber for a period of 2 h per day to normoxia while the pups were kept in the hyperoxia chamber. Cages were cleaned quickly during this interruption to hyperoxia. Oxygen concentration in the chamber returned to 90% level 15 min after the chamber was opened to remove or return the mother to hyperoxia chamber. Normoxic pups were kept in a similar chamber with 21% O_2_ exposure. Bacterial LPS (*E. coli*, 0111:B4, sigma, St. Louis, MO, USA) was suspended in sterile pyrogen-free 0.9% saline. A dose of 10 mg LPS or equal volume of saline (control) was injected IP into rat pups at 7d postnatal age. The pups were allowed to continue under hyperoxia (or normoxic) condition for three additional days after the IP injection of LPS or saline.

### 2.2. Freezing protocol and embedding

At 10d postnatal age, pups were removed and anesthetized briefly with inhalation of 1–2% isoflurane. The rat pups were then quickly decapitated and lungs were harvested quickly. The blood was washed from lungs by repeated infusion of Hank’s Buffered salt solution into the pulmonary artery, after which the lungs were frozen for cryoimaging as described below.

Lung tissue metabolic state was preserved by rapid freezing in chilled isopentane and then in liquid nitrogen (LN_2_) followed by storage in LN_2_. The entire process of harvesting and preserving lung tissue in LN_2_ from the time of euthanizing the rat pup was completed within 10 min.

For fluorescence imaging, the frozen lung was embedded in a customized black mounting medium (not fluorescent in the wavelengths of interest) and placed on a chilled aluminum plate to keep the tissue in place for freezing and slicing. The embedding process started with freezing the base medium, embedding the frozen tissue and fixing its position and then storage in an ultralow freezer (−80°C) prior to imaging.

### 2.3. Cryoimager

**Fig. 1:**
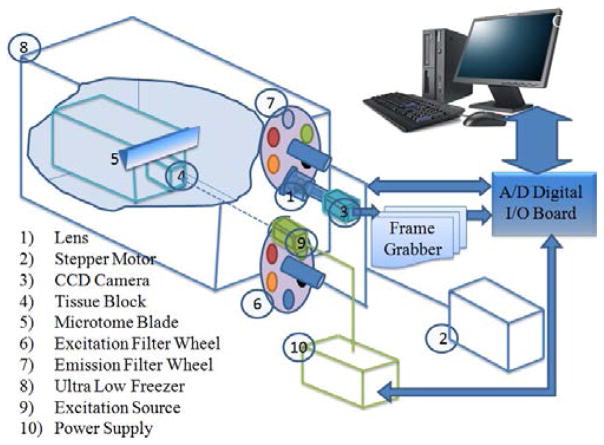
Schematic of cryoimager.^33^

Figure 1 shows the schematic for the 3D cryoimager used in this study. The cryoimager is an automated image acquisition and analysis system consisting of software and hardware designed to acquire fluorescence images of tissue sections. A motor-driven microtome sequentially sections’ frozen tissue at the desired slice thickness while filtered light from a mercury arc lamp excites up to five distinct fluorophores in the exposed surface of the tissue block. The excitation light source is a 200 W mercury arc lamp filtered at the excitation wavelength of NADH and FAD. The excitation band-pass filter used for NADH is 350 nm (80 nm bandwidth, UV Pass Blacklite, HD Dichroic, Los Angeles, CA) and that for FAD is 437 nm (20 nm bandwidth, 440QV21, Omega Optical, Brattleboro, VT) and the emission filter for NADH is 460 nm (50 nm bandwidth, D460/50 M, Chroma, Bellows Falls, VT) and that for FAD is 537 nm (50 nm bandwidth, QMAX EM 510–560, Omega Optical, Brattleboro, VT). At each slice, a CCD camera records a fluorescence image of the tissue block in pixel dimensions of 30 × 30 *μ*m to be later analyzed for fluorophore distribution. The microtome is housed in a freezer unit that maintains the sample at −80°C during sample slicing and image acquisition. The resolution in the *z*-direction of microtome slices can be as small as 5 *μ*m. For this study, we used a resolution of 30 *μ*m in the *z*-direction, which resulted in ~ 700 *z*-slices per lung.^33,43,44^

### 2.4. Calibration

A calibration method was designed to compensate for day-to-day variation of light intensity and nonuniformity of the illumination pattern. At the beginning of each experiment and before slicing the tissue, a uniform fluorescent flat plate was placed in the same position as tissue and imaged in all channels to acquire the illumination pattern. Since the fluorescence of the standard is in both the NADH and FAD channels, it also accounts for day-to-day light intensity and uniformity changes in all channels. All the images in each channel were then normalized by dividing each image to the flat plate image, captured in the same channel.

### 2.5. Data analysis

FAD and NADH autofluorescence images (containing 700 slices per lung) from each group of lungs were processed using MATLAB (The MathWorks, Inc., Natick, MA). The composite images were created using all the image slices for each lung, for both NADH and FAD signals. The ratio of NADH and FAD, known as the NADH RR,^33^ was calculated voxel by voxel, using MATLAB, according to Eq. (1).

(1) NADHRaedoxRatio=NADHRR=NADH/FAD.

For each lung, a histogram of RR values was created, and the first moment of this histogram (mean value of the NADH redox) was calculated for the whole volume of the tissue according to Eq. (2).

(2) $$\text{Mean}=\frac{1}{N_{x}\times N_{y}\times N_{z}}\times \sum_{i=1}^{N_{x}}\sum_{j=1}^{N_{y}}\sum_{k=1}^{N_{z}}\text{Lung\_Volume}(i,j,k).$$

Here *N_x_*, *N_y_* and *N_z_* are the number of pixels in the *x*-, *y*- and *z*- directions and the pixel size is 30 *μ*m in all directions. The histograms were calculated for quantitative comparison between normoxic + KCN, normoxic lungs, normoxic + LPS hyperoxic and hyperoxic + LPS. Statistical comparisons were carried out using ANOVA followed by Tukey’s test, with *p* < 0.05 as the criterion for statistical significance.

## 3. Results

**Fig. 2:**
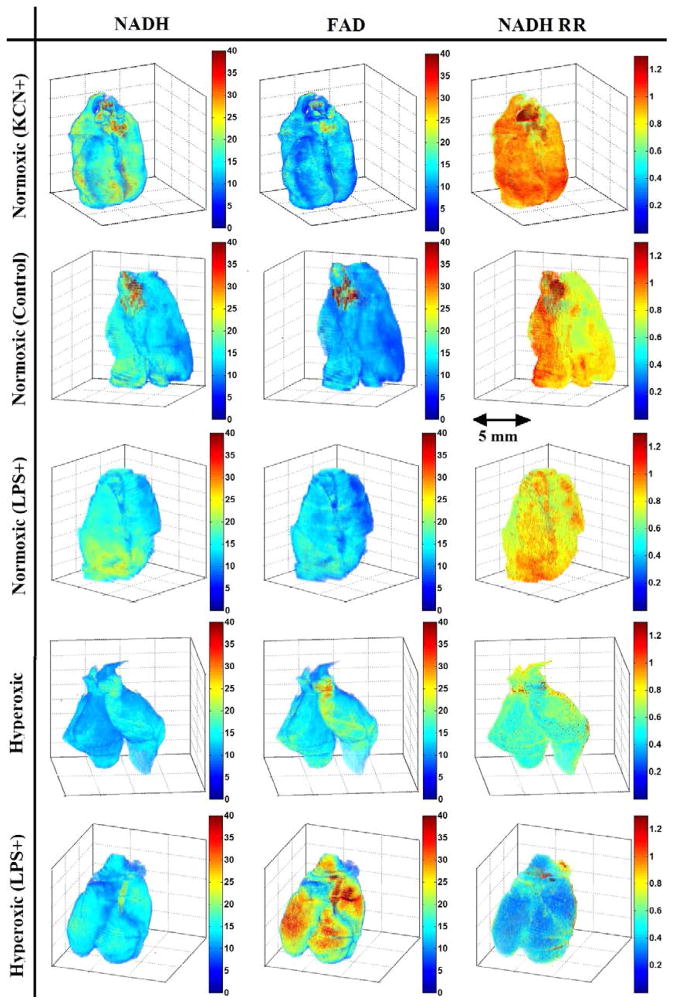
Volume rendering of NADH, FAD and NADH redox of a representative lung in each group.

**Fig. 3:**
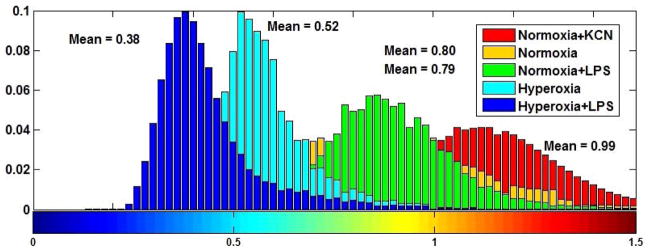
NADH redox histograms for a representative lung in each group.

As described, the fluorescence images of NADH and FAD were used in the calculation of NADH RR. Figure 2 shows the 3D volume rendering of NADH, FAD and NADH redox for a representative lung from each of the normoxic + KCN, normoxic, normoxic + LPS, hyperoxic and hyperoxic + LPS lung groups, in decreasing order of RR, and Fig. 3 shows the NADH redox histograms of the representative lungs presented in Fig. 2. The normoxic lungs treated with KCN show the highest levels of NADH RR compared to other lungs, establishing an upper limit for NADH RR in this study. The lungs in the normoxic and normoxia + LPS group (second and third rows in Fig. 2) show a lower concentration of FAD and a higher concentration of NADH compared to hyperoxic and hyperoxia + LPS groups (fourth and fifth row in Fig. 2). Thus, the NADH RR is lower (more oxidized) in the hyperoxia +/− LPS groups compared to the normoxic +/− LPS groups.

**Fig. 4:**
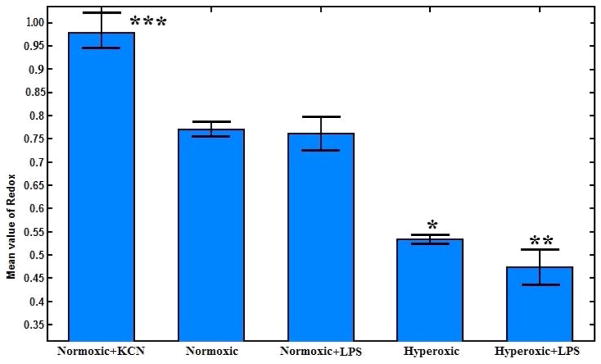
Bar graph showing the mean and standard errors of the mean value of mitochondrial RR for each of the five groups of lungs. The number of lungs N = 3, 5, 4, 4 and 4 for normoxic + KCN, normoxic, normoxic + LPS, hyperoxic and hyperoxic + LPS, respectively. The results show a significant difference between normoxic and hyperoxic (*p < 0.01), normoxic and hyperoxic + LPS (**p < 0.01), and normoxic and normoxic + KCN lungs (***p < 0.01).

The mean values of NADH RR for all the lungs are shown in a bar graph including the standard errors, in Fig. 4. KCN perfused lungs showed the highest NADH redox (mean value of ~ 0.98), which is 29% more reduced than that for normoxic lungs (mean value of 0.77). Comparing the normoxic lungs to normoxic + LPS lungs (NADH redox mean value of 0.76), the results show no significant change in the biochemical state of the tissue, which indicates that stimulating the lung with LPS has no significant effect on the redox status of the respiratory chain of the lung. NADH RR values for hyperoxic + LPS lungs (mean value of 0.47) and hyperoxic lungs (mean value of 0.53) were not significantly different. Thus, comparing the results for all lungs (hyperoxic + LPS +/− versus normoxic LPS +/−), it can be concluded that there is a higher level of oxidative stress in the hyperoxic + LPS lung versus the normal + LPS lung compared to their controls (hyperoxic and normoxic lungs, respectively). Comparing the mean values of normoxic versus hyperoxic lung, it can be seen that the NADH RR indicates a 31% more oxidized chain in hyperoxic lungs compared with normoxic lungs.

## 4. Discussion and Conclusion

Oxygen therapy has been used in clinical medicine for many years despite the recognition of pulmonary oxygen toxicity as a problem for nearly 70 years. Cellular metabolism under hyperoxic conditions leads to an increase in the rate of formation of oxygen free radicals and results in oxygen-induced lung injury. Additionally, LPS exposure is a common factor involved in ALI and results in endothelial apoptosis and mitochondrial dysfunction.

In this study, an optical imaging technique was used to probe lung tissue mitochondrial redox state and energy homeostasis in the intact functioning lungs, to evaluate the effect of LPS as well as exposure to high O_2_ concentrations. Bacterial LPS is a major factor in causing ALI and induces the release of ROS, which in turn may lead to higher oxidative stress in the lung tissue. Hyperoxia leads to enhanced production of ROS, which in turn causes oxidative cellular injury and disruption of lung function. Thus, NADH RR can be used as a quantitative marker to evaluate oxidative stress in diseased and normal lung tissue.

In order to show the dynamic range of mitochondrial redox state, recently,^20^ we demonstrated changes in the RR of a normoxic adult rat lung when perfused with KCN (potassium cyanide), as a negative control (the most reduced state). KCN is a complex IV inhibitor of the respiratory chain which reduces the chain, and hence provides an upper boundary on NADH RR.^20,42,45^ KCN was chosen since in a previous study,^45^ comparing perfusion with KCN, anoxia (95% N_2_ and 5% CO_2_) and CO (95% CO, 5% CO_2_), the KCN group showed the largest inhibitory effect on the redox state of the lung tissue. In the present study, hyperoxia and KCN had the opposite effects on lung NADH RR, which was 29% higher (more reduced) in KCN + normoxic lungs and 49% lower (more oxidized) in hyperoxic lungs as compared to that in normoxic lungs.^20^

Our hypothesis is that LPS accentuates a pre-existing insult to lungs caused by hyperoxia. In this work, the *in situ* assessment of the oxidative state in the LPS-induced and hyperoxic lung injury model was demonstrated using optical cryoimaging of intact organ. Here, NADH RR was used as a quantitative marker of measuring the oxidative stress induced by hyperoxia and LPS stimulation in rat lung tissue. We initially confirmed the utility of the methodology in our previous studies.^20,33^ In the presence of more O_2_, NADH and FADH2 are found in their oxidized form (NAD, FAD). Therefore, in hyperoxic and hyperoxic + LPS conditions, we expect to have higher concentrations of FAD and lower concentrations of NADH compared to normoxic and normoxic + LPS groups. As seen from the images in Fig. 2, the FAD concentration is higher (brighter images) and NADH concentration is lower (darker images) in the last two rows compared to the first three. Comparing the histograms showing the mean values for normal lung induced by LPS and its control (normal) reveals that LPS stimulation did not affect the redox status of mitochondrial respiratory chain of normoxic or hyperoxic lungs.

Another important conclusion is the effect of oxygen on the rat lung tissue apart from the LPS injection. Exposing the lung tissue to higher concentrations of oxygen causes lung injury from O_2_ toxicity. This injury occurs as a result of enhanced production of ROS, which may further impair lung function. As can be seen from the images in Fig. 2, in the hyperoxic group, the FAD concentration is increased and the NADH concentration is decreased compared to the normoxic lung. Thus, the NADH RR of hyperoxic lung is less than the normoxic lung, which shows a more oxidized mitochondrial respiratory chain in the hyperoxic lung compared to the normoxic lung. Although the redox state of mitochondrial respiratory chain does not directly measure ROS, our observation suggests a possible mechanism for higher prevalence of lung diseases with the use of high concentrations of supplemental oxygen.

Our results demonstrate the utility of optical cryoimaging for measuring lung tissue mitochondrial redox state in four different conditions. The NADH RR reveals differences in tissue NADH, FAD and RR signals between hyperoxic + LPS versus hyperoxic rat lungs as well as hyperoxic rat versus normoxic group. Furthermore, the research presented here sets the stage for future studies using *in vivo* surface fluorescence imaging of the mitochondrial signals of lungs from hyperoxic rats to follow the mitochondrial redox state and potentially monitor the efficacy of therapeutic regimens. The limitation of translation to *in vivo* and optical surface fluorescence imaging is that it may not detect changes deeper than 500 *μ*m (the imaging depth is normally around 200–300 *μ*m). However, this resolution is more than sufficient for determining the RR of homogeneous parenchymal tissue, which has a thickness (air to plasma) of 1.6 *μ*m^47^ for lung tissue.
